# Comparative acute efficacy and tolerability of OROS and immediate release formulations of methylphenidate in the treatment of adults with attention-deficit/hyperactivity disorder

**DOI:** 10.1186/1471-244X-7-49

**Published:** 2007-09-14

**Authors:** Joseph Biederman, Eric O Mick, Craig Surman, Robert Doyle, Paul Hammerness, Evan Michel, Jessica Martin, Thomas J Spencer

**Affiliations:** 1 Clinical and Research Program in Pediatric Psychopharmacology and Adult ADHD at the Massachusetts General Hospital, Boston, MA 02114, USA; 2 Department of Psychiatry at Harvard Medical School, Boston, MA., USA

## Abstract

**Background:**

The main aim of this study was to compare the safety and efficacy of IR MPH administered three times daily to those of once daily OROS-MPH.

**Methods:**

Subjects were outpatient adults satisfying full diagnostic criteria for DSM-IV ADHD between 19 and 60 years of age. Data from two independently conducted 6-week placebo controlled, randomized clinical trials of IR-MPH (tid) and of OROS-MPH were pooled to create three study groups: Placebo (N = 116), IR-MPH (tid) (N = 102) and OROS-MPH (N = 67).

**Results:**

Eight-five percent (N = 99) of placebo treated subjects, 77% (N = 79) of the IR-MPH (tid) treated subjects, and 82% (N = 55) of the OROS-MPH treated subjects completed the 6-week trial. Total daily doses at endpoint were 80.9 ± 31.9 mg, 74.8 ± 26.2 mg, and 95.4 ± 26.3 mg in the OROS-MPH, IR-MPH (tid), and placebo groups, respectively. At endpoint, 66% (N = 44) of subjects receiving OROS-MPH and 70% (N = 71) of subjects receiving IR-MPH (tid) were considered responders compared with 31% (N = 36) on placebo.

**Conclusion:**

Comparison of data from two similarly designed, large, randomized, placebo-controlled, trials, showed that equipotent daily doses of once daily OROS-MPH had similar efficacy to that of TID administered IR MPH.

**Trial Registration:**

The trial of OROS-MPH was registered at clinicaltrials.gov, number NCT00181571.

## Background

Attention deficit hyperactivity disorder (ADHD) is a persistent disorder associated with high levels of morbidity and disability across the lifecycle. Although long conceptualized as a pediatric disorder, it is now estimated to affect between 3%–5% of adults in this country [1,2]. The extant literature documents several similarities between adult and pediatric ADHD in terms of psychiatric comorbidity and neuropsychological deficits, as well as a neural and genetic underpinning that supports the syndromatic continuity of ADHD across the lifecycle [3]. Recent work has also documented the severe functional impairments associated with ADHD including educational and occupational under attainment, driving accidents, addictive behaviors and a wide range of interpersonal deficits adversely impacting all aspects of life [4] supporting the need for the identification of safe and effective treatments for this disorder in adults.

While methylphenidate (MPH) remains one of the leading pharmacological treatments of pediatric ADHD, there is a limited literature on its safety and efficacy in the treatment of adults with ADHD. In contrast to equivocal results observed in early studies that used daily doses of approximately 0.5 mg/kg of immediate release methylphenidate (IR MPH) [5-9], clearer patterns of response were documented in studies using doses of approximately 1 mg kg of IR-MPH [10,11]. These findings suggest that MPH is effective in the treatment of adults with ADHD when used in weight adjusted doses equipotent to those used in pediatrics.

Results from a recent, large randomized, six week, placebocontrolled clinical trial documented that osmotic release methylphenidate (OROS-MPH) at daily average doses of 1 mg/kg/day was also highly effective and well tolerated in the treatment of adults with ADHD [12]. Since Spencer et al [11] administered MPH three times daily and the long acting formulation of OROS-MPH was designed to provide day-long coverage, these results suggest that a key component of efficacy observed in these studies may also be day long pharmacological coverage. Although OROS-MPH was designed to mimic the pharmacokinetic profile of IR MPH administered three times daily, is unclear if these two formulations of MPH are equally tolerated and effective since there have been no head to head comparisons conducted.

In the absence of randomized head to head comparisons, existing studies that used OROS and IR formulations of MPH could be compared if they employed similar methodology. We have conducted two six week, randomized, placebo-controlled studies of OROS-MPH and IR MPH that used nearly identical dosing and assessment methodology. The main aim of this study was to compare the safety and efficacy of equipotent doses of IR MPH administered TID to those of once daily OROS-MPH. To this end we used data from two similarly designed, large, randomized, placebo-controlled, six week trials of IR and OROS-MPH in adults with DSM-IV ADHD [11,12]. We hypothesized that once daily OROS-MPH would show similar efficacy and tolerability to that of IR-MPH administered three times a day.

## Methods

### Subjects

Subjects in both studies were outpatient adults with ADHD between 19 and 60 years of age. To be included, subjects had to satisfy full diagnostic criteria for DSM-IV ADHD based on clinical assessment and confirmed by structured diagnostic interview. There was no overlap in participation between the study samples. We excluded potential subjects if they had clinically significant chronic medical conditions, abnormal baseline laboratory values, I.Q. <80, delirium, dementia, or amnestic disorders, other clinically unstable psychiatric conditions (i.e., bipolar disorder, psychosis, suicidality), drug or alcohol abuse or dependence within the six months preceding the study, or previous adequate trial of methylphenidate. We also excluded pregnant or nursing females. The human research committee of the institutional review board (IRB) approved these studies and all subjects completed a written informed consent.

### Procedures

#### Randomized Trial of IR-MPH (tid) [11]

This was a double-blind, randomized, 6 week, placebo-controlled, parallel design study of MPH in the treatment of adult ADHD. Patients were randomized to MPH or placebo at a ratio of 2.5:1. Weekly supplies of MPH or placebo were dispensed by the pharmacy in identically appearing 5 and10 mg capsules. Study physicians prescribed medication under double blind conditions in three times per day dosing (7:30 am, noon and 5 pm). Study medication was titrated (forced titration) up to 0.5 mg/kg/day by weekone, 0.75 mg/kg/day by week two and 1.0 mg/kg/day by week three, in TID dosing, unless adverse effects emerged. The dose could have been increased to a maximum of 1.3 mg/kg by weeks 5 and 6 if efficacy was partial and treatment was well tolerated. Other psychoactive medications were not permitted during the protocol.

#### Randomized Trial of OROS-MPH [12]

This was a double blind, randomized, 6-week, placebo-controlled, parallel design study of OROS-MPH. Patients were randomized to OROS-MPH or placebo at a ratio of 1:1. Medication was titrated to optimal response (a maximum daily dose of 1.3 mg/kg; initial dose of 36 mg). During titration to optimal response, dose was increased by 36 mg/day for only those subjects who failed to attain an *a priori *definition of improvement defined by a clinical global impressions scale-improvement (CGI-Improvement) score of 1 or 2 and a reduction in the Adult ADHD Investigator Symptom Rating Scale (AISRS) score larger than 30%) and who did not experience adverse effects. All doses of OROS-MPH and placebo were delivered in identically appearing tablets.

### Assessment

To assess inclusion and exclusion criteria, all subjects underwent a comprehensive clinical assessment which included a psychiatric evaluation by a board certified psychiatrist, structured diagnostic interview, medical history, vital signs, and laboratory assessments (liver function tests, complete blood count, and electrocardiogram). The structured diagnostic interview used was the Structured Clinical Interview for DSM-IV (SCID) [13], supplemented for childhood disorders by modules (DSM-IV ADHD and conduct disorder) from the Kiddie SADSE (Epidemiologic Version). [14]. This interview was selected because it diagnoses both lifetime and current month psychopathology and has been used extensively in clinical and research settings [11,15].

To have been given a full diagnosis of adult ADHD, the subject must have: a)met full DSMIVR criteria (at least 6 of 9 symptoms) for inattentive and/or hyperactive/impulsive subtypes [16] by the age of seven as well as within the past month (i.e. ADHD-IA, ADHD-HI and ADHD-C subjects were enrolled); b)described a chronic course of ADHD symptomatology from childhood to adulthood and c) endorsed a moderate or severe level of impairment attributed to the ADHD symptoms.

Overall severity and change in severity of ADHD was assessed with the Clinical Global Impression Scale (CGI) [17]. The CGI includes Global Severity (1 = not ill, to 7 = extremely ill) and Global Improvement (1 = very much improved, to 7 = very much worse) Scales. The Adult ADHD Investigator System report Scale (AISRS) [18] was used to assess each of the 18 individual criteria symptoms of ADHD in DSMIV on a severity grid (0 = not present; 3 = severe; overall minimum score = 0; maximum score = 54). To assess symptoms of depression and anxiety, we used the 17-item Hamilton Depression Scale (HAM-D, minimum = 0; max = 52) [19] and the Hamilton Anxiety Scale (HAM-A, minimum = 0; max = 56)[20]. A global measure of psychosocial functioning (global assessment of functioning (GAF) scale) was rated according to guidelines in DSM-IV [21]. Adverse events were elicited by spontaneous reports through open-ended questions at each visit. Weight and vital signs were obtained at each visit and an EKG was performed at baseline and endpoint. Both raters and subjects were blind to treatment assignment.

### Statistical Analysis

Analyses were intention to treat (ITT) with the exception that subjects must have been assessed on drug or placebo for at least one week (92% [11] and 95% [12] of randomized returned for at least 1 assessment). A mixed-effects model repeated measures approach was used to account for missing data in our longitudinal assessments of safety (i.e. weight and vital signs) and efficacy. Models assessing symptom improvement of the primary measure of outcome (AISRS score) were adjusted for any demographic or clinical differences at baseline between the groups and baseline AISRS score. Omnibus and pair wise comparisons were made with post estimation Wald tests such that χ^2 ^statistics are reported for continuous data. Continuous and categorical data were tested with ANOVA and Pearson's χ^2^, respectively for non-longitudinal data (i.e. demographics at baseline, prevalence of adverse effects or response at endpoint, etc). Statistical significance was determined at p < 0.05. For simplicity of exposition, placebo subjects from both studies were pooled into a single placebo group. Thus, three groups were compared: Placebo (N = 116), IR-MPH (tid) (N = 102) and OROS-MPH (N = 67).

## Results

Clinical and demographic characteristics are presented in Table 1. Although there were small but statistically significant differences in age between the groups (statistically significant for OROS-MPH versus placebo), each of the three groups were in their mid thirties, on average. There were no statistically significant differences in gender, ADHD age at onset, number of symptoms, or clinical impression of severity at baseline.

**Table 1:** Demographic and Clinical Characteristics at Baseline

	**Placebo N = 116**	**IR-MPH (tid) N = 102**	**OROS-MPH N = 67**	
		
	mean ± sd/N (%)	mean ± sd/N (%)	mean ± sd/N (%)	Omnibus Statistic
	
Age (years)	38.5 ± 9.1	35.7 ± 9.8	32.7 ± 18.5 *	F(2,282) = 5.0, p = 0.007
Sex (male)	58 (50)	60 (59)	38 (57)	χ^2^_(2) _= 1.8, p = 0.4
Weight (kg)	82.9 ± 18.9	79.9 ± 15.8	84.1 ± 20.1	F(2,282) = 1.3, p = 0.3
ADHD				
Onset (years)	5.0 ± 2.9	6.3 ± 6.7	5.2 ± 2.2	F(2,253) = 2.0, p = 0.1
Symptoms (lifetime)	14.3 ± 2.8	13.5 ± 3.4	14.4 ± 2.7	F(2,248) = 4.3, p = 0.1
Symptoms (Current)	12.1 ± 3.7	11.5 ± 3.9	12.2 ± 2.9	F(2,248) = 2.0, p = 0.4
CGI Severity				
Mild	1 (1)	1 (1)	1 (1)	χ^2^_(6) _= 9.6, p = 0.1
Moderate	52 (45)	31 (30)	27 (40)	
Marked	53 (46)	60 (59)	38 (57)	
Severe	10 (9)	10 (10)	1 (1)	
GAF				
Past (worst)	52.2 ± 5.6	52.3 ± 5.9	51.4 ± 4.7	F(2,259) = 0.6, p = 0.5
Current	59.0 ± 4.9	59.9 ± 5.0	57.8 ± 4.2^$^	F(2,259) = 4.1, p = 0.02
HAM-A	4.6 ± 3.9	5.8 ± 4.8	4.4 ± 3.8	F(2,281) = 3.2, p = 0.04
HAM-D	4.4 ± 4.5	5.1 ± 5.3	4.8 ± 4.7	F(2.282) = 0.6, p = 0.6

*, p < 0.05 versus Placebo, $, p < 0.05 versus IR-MPH (tid).

**Figure 1:**
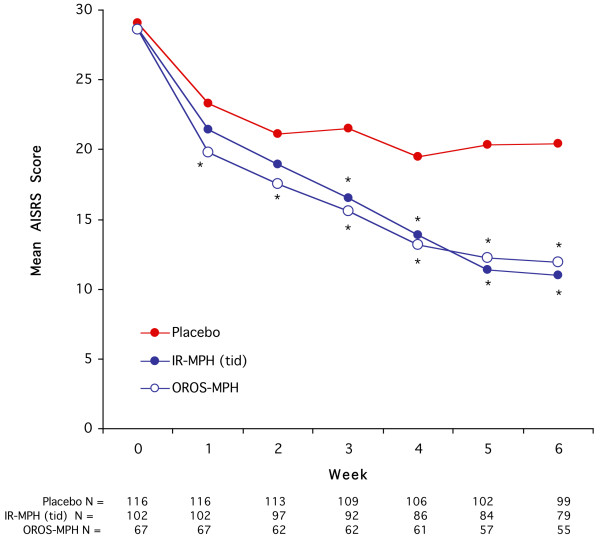
Clinical Ratings of ADHD Symptoms.

At baseline, there were small statistically significant but not clinically meaningful differences in global assessment of functioning and ratings of anxiety that were slightly worse in the OROS-MPH than in the IR MPH groups (Table 1). Also, there was a small but significant difference in the AISRS score between the three groups (F(2.282) = 4.1, p = 0.02) that was accounted for by a difference between the OROS-MPH (30.1 ± 5.9) and the placebo (32.1 ± 7.9) subjects (Figure 1).

There were no differences in dose at endpoint between IR-MPH (tid) and OROS-MPH (0.97 ± 0.21 mg/kg versus 0.99 ± 0.32 mg/kg; p = 0.09) but both were statistically significantly lower than placebo (1.15 ± 0.21 mg/kg; p = 0.001). Total daily doses at endpoint were 80.9 ± 31.9 mg, 74.8 ± 26.2 mg, and 95.4 ± 26.3 mg in the OROS-MPH, IR-MPH (tid), and placebo groups, respectively.

Eight-five percent (N = 98) of placebo treated subjects, 75% (N = 76) of the IR-MPH (tid) treated subjects, and 81% (N = 54) of the OROS-MPH treated subjects completed the 6-week trial (χ^2^_(2) _= 2.3, p = 0.3). In placebo, IR-MPH (tid) and OROS-MPH subjects the reasons for dropout were: adverse effects (N = 5, N = 14, and N = 9, respectively), lost to follow-up (N = 3, N = 3, and N = 4, respectively), procedural/lack of compliance (N = 6, N = 9 and N = 0, respectively), and lack of effect (N = 4, N = 0, and N = 0, respectively).

Adjusting for baseline AISRS value, there were statistically significant treatment effects compared to placebo for both the IR-MPH (tid) (Wald χ^2^_(6) _= 40.9, p < 0.0001) and the OROS-MPH (Wald χ^2^_(6) _= 23.7, p = 0.0006) groups. There was not a statistically (Wald χ^2^_(6) _= 7.8, p = 0.3) or clinically significant difference between the IR-MPH (tid) and OROS-MPH treated subjects (Figure 1).

**Figure 2:**
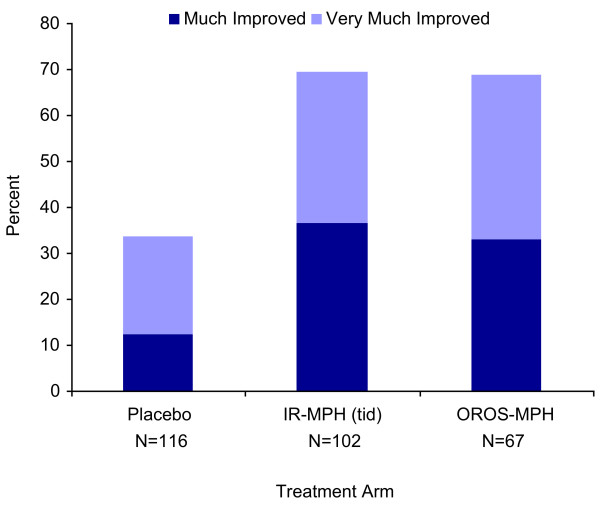
Clinical Ratings of Improvement.

The rate of improvement according to the psychiatrist rated clinical global impression (CGI-I) for ADHD was statistically significantly higher for both IR-MPH (tid) (χ^2^_(1) _= 27.7, p < 0.001) and OROS-MPH (χ^2^_(1) _= 21.2, p < 0.001) groups compared with placebo (Figure 2), and there were no statistically (χ^2^_(1) _= 0.008, p = 0.9) or clinically significant differences between the two formulations of MPH. Forty percent (N = 46), 80% (N = 80), and 69% (N = 46) of the placebo, IR-MPH (tid) and OROS-MPH groups, respectively attained a 30% reduction of baseline AISRS scores at endpoint. Both the IR-MPH (tid) and the OROS-MPH groups were statistically significantly more likely to have a 30% reduction in symptoms than placebo (p < 0.001) and were not different from one another (p = 0.1).

At endpoint, 66% (N = 44) of subjects receiving OROS-MPH and 70% (N = 71) of subjects receiving IR-MPH (tid) were considered responders compared with 31% (N = 36) on placebo (χ^2^(2) = 38.1, p < 0.001), using our *a priori *definition of response of much or very much improved on the CGI-I *plus *more than a 30% reduction in symptoms on the AISRS. Both active medication groups were statistically significantly more likely to demonstrate this level of improvement compared with placebo (p < 0.001) but not when compared to one another (p = 0.6).

The rate of adverse effects reported over the study period is presented in Table 2. Both the IR-MPH (tid) and the OROS-MPH treated subjects were more likely to report dry mouth, decreased appetite, sleep difficulties and moodiness than were subjects treated with placebo (Table 2). There was a statistically significant greater weight loss in the IR-MPH (tid) (-2.1 ± 2.4 kg, p < 0.001) and the OROS-MPH groups (-2.8 ± 1.9 kg, p < 0.001) than in the placebo group(0.02 ± 1.7 kg); differences in weight loss between OROS-MPH and IR-MPH (tid) were small but statistically significant (p = 0.03). In addition subjects treated with OROS-MPH were more likely than subjects treated with IR-MPH (tid) to report having dry mouth and decreased appetite (Table 2). Complaints of GI difficulties were statistically significantly elevated in the OROS-MPH subjects only.

**Table 2:** Adverse Effects

	**Placebo N = 116**	**IR-MPH (tid) N = 102**	**OROS-MPH N = 67**	
		
	N (%)	N (%)	N (%)	Omnibus Statistic
	
Dry Mouth	5 (4)	22 (22) *	25 (37)^*,$^	χ^2^_(2) _= 32.2, p < 0.001
Headache	30 (26)	22 (22)	24 (36)	χ^2^_(2) _= 4.2, p = 0.1
Decreased Appetite	4 (3)	11 (11) *	24 (36)^*,$^	χ^2^_(2) _= 38.8, p < 0.001
GI Complaints	13 (11)	17 (17)	20 (29)^*,$^	χ^2^_(2) _= 10.3, p = 0.006
Sleep Problems	6 (5)	14 (14) *	14 (21) *	χ^2^_(2) _= 10.5, p = 0.005
Moodiness	7 (6)	15 (15) *	12 (18) *	χ^2^_(2) _= 6.9, p = 0.03
Aches and Pains	13 (11)	7 (7)	9 (13)	χ^2^_(2) _= 2.1, p = 0.3

*, p < 0.05 versus Placebo, $, p < 0.05 versus IR-MPH (tid)

There were no serious adverse events reported. Specific adverse effects leading to drop out in the placebo group were irritability (N = 1), fatigue (N = 1), increased pulse/racing heart (N = 1) and elevated blood pressure (N = 2). Adverse effects leading to dropout in the IR-MPH subjects were jitteriness (N = 2), irritability (N = 2), depression, (N = 1), anxiety (N = 1), over-focus (N = 1), headache (N = 1), insomnia (N = 1), and elevated blood pressure (N = 5). Adverse effects leading to dropout in the OROS-MPH groups were jitteriness (N = 1), irritability (N = 3), depression (N = 1), anxiety (N = 1), increased pulse/racing heart (N = 2) and elevated blood pressure (N = 1).

Changes from baseline to endpoint on cardiac measures are presented in Table 3. As expected, there were small but statistically significant differences in diastolic blood pressure and pulse between both active treatment groups and placebo subjects. There were no differences between the IR-MPH (tid) and the OROS-MPH groups with the exception of a smaller increase in pulse in OROS-MPH group that, although statistically significant (p = 0.049), would be of limited clinical significance. Outliers analysis of cardiovascular data revealed that significantly more subjects treated with OROS-MPH and IR-MPH (tid) attained clinically significant elevated heart rate (>100 bpm: 6 (9%) and 3 (3%) vs 1 (1%) respectively, χ^2^_(2) _= 8.5, p = 0.02; maximum value observed was 114 bpm). Other cardiovascular outliers were not significantly more likely to occur in subjects treated with active medication than with placebo: systolic blood pressure (>140 mmHg: 5 (8%) and 14 (14%) vs. 6 (5%), χ^2^_(2) _= 5.5, p = 0.06; maximum value observed was 156 mmHg), diastolic blood pressure (>90 mmHg: 2 (3%) and 8 (8%) vs. 3 (3%), χ^2^_(1) _= 4.1, p = 0.1; maximum value observed was 102), or QTC interval (>460 msecs: 1 (2%) and 1 (2%) vs. 2 (2%), χ^2^_(1) _= 0.1, p = 0.9; maximum value observed was 488 msecs). For none of these clinical outliers was the difference between IR-MPH (tid) and OROS-MPH statistically significant.

**Table 3:** Cardiac Parameters and Baseline and Endpoint

	**Placebo N = 116**	**IR-MPH (tid) N = 102**	**OROS-MPH N = 67**	
		
	mean ± sd	mean ± sd	mean ± sd	Omnibus Statistic
	
**Systolic Blood Pressure**				
Baseline	121.4 ± 13.7	125.6 ± 13.3	119.2 ± 13.2	
Endpoint	120.3 ± 13.0	127.5 ± 12.7	122.6 ± 11.9	
Change	-1.2 ± 12.3	2.0 ± 12.6	3.5 ± 11.8	χ^2^_(2) _= 6.7, p = 0.03
**Diastolic Blood Pressure**				
Baseline	71.3 ± 10.5	75.1 ± 9.6	68.6 ± 8.9	
Endpoint	69.8 ± 10.0	77.3 ± 9.2	72.8 ± 9.3	
Change	-1.5 ± 8.4	2.1 ± 8.8 *	4.0 ± 8.5 *	χ^2^_(2) _= 19.9, p < 0.0001
**Pulse**				
Baseline	76.2 ± 10.9	76.5 ± 12.5	78.2 ± 11.6	
Endpoint	71.5 ± 10.9	82.3 ± 12.7	82.9 ± 12.6	
Change	-4.8 ± 11.7	5.9 ± 12.9*	4.5 ± 10.5^*,$^	χ^2^_(2) _= 27.9, p < 0.0001
**PR Interval**				
Baseline	153.2 ± 18.7	151.3 ± 25.8	151.3 ± 18.3	
Endpoint	151.8 ± 17.8	147.9 ± 20.4	147.9 ± 21.1	
Change	-1.1 ± 13.7	-2.5 ± 11.8	-1.2 ± 11.3	χ^2^_(2) _= 0.6, p = 0.8
**QRS Interval**				
Baseline	87.5 ± 10.9	90.1 ± 12.8	93.3 ± 9.9	
Endpoint	88.1 ± 10.4	87.8 ± 10.9	91.5 ± 11.3	
Change	0.5 ± 8.6	-1.2 ± 9.7	-1.2 ± 5.8	χ^2^_(2) _= 3.1, p = 0.2
**QTc Interval@**				
Baseline	413.4 ± 18.0	417.1 ± 19.8	412.5 ± 15.6	
Endpoint	413.6 ± 16.3	420.9 ± 18.4	414.5 ± 18.8	
Change	0.5 ± 18.4	6.1 ± 16.0	1.9 ± 15.7	χ^2^_(2) _= 2.7, p = 0.3

*, p < 0.05 versus Placebo, **$**, p < 0.05 versus IR-MPH (tid), **@**, Bazzett's Correction

## Discussion

Comparison of data from two similarly designed, large, randomized, placebo-controlled, 6-week trials of IR and OROS-MPH in adults with DSM-IV ADHD, showed that once daily OROS-MPH had similar efficacy and tolerability to that of equipotent daily doses of IR-MPH administered three times per day. These results confirmed the study hypothesis that once daily OROS-MPH is as effective as three times daily IR MPH at similar doses. The finding that the same daily dose of once daily OROS-MPH was as effective as that of IR-MPH (tid) is consistent with the pharmacokinetic profile of OROS-MPH that was formulated to deliver three doses of IR MPH across the day with once daily administration.

The magnitude of response observed in this study is consistent with results reported in over 250 controlled trials of stimulants in pediatric ADHD using similar weight corrected daily doses of 1 mg/kg/day. Moreover, the response rate of 70% observed for both formulations of MPH in adults with ADHD at daily doses of 1 mg/kg are much higher than the average 52% response rate reported in early studies of MPH using average daily doses of 0.5 mg/kg [5-9]. Taken together, these results support the hypothesis that daily doses of 1 mg/kg/day are needed to attain a robust response to MPH in the treatment of adults with ADHD.

Despite the relatively high daily doses of MPH used, both formulations of MPH were well tolerated as manifested in the high completion rate (~ 80% for both formulations) and absence of serious adverse events. While subjects treated with OROS-MPH formulation more commonly reported dry mouth, decreased appetite, and GI complaints than subjects treated with IR-MPH (tid), only 14% of each group dropped from the study due to adverse effects. Treatment with both formulations of MPH was associated with similarly small but statistically significant effects in diastolic blood pressure and pulse relative to placebo. Although abnormal values in cardiovascular parameters (maximum values observed were 114 bpm for heart rate, 156 mmHg for systolic blood pressure, 102 mmHg for diastolic blood pressure, and 488 msecs for QTc interval) were not associated with medical complications in this study, adults with ADHD should be monitored for changes in blood pressure and weight loss when receiving treatment with MPH.

The documentation that once daily OROS-MPH is as effective as IR MPH administered three times daily has important clinical implications. Because adults with ADHD tend to be forgetful, once daily administration of OROS-MPH may lead to better compliance. Also, since the delivery of MPH via OROS is gradual and the MPH cannot be as easily extracted from the OROS tablet this formulation has a lower abuse potential than IR-MPH. This is so because the abuse potential of MPH is thought to be due to the rapid onset of blockade of the presynaptic dopamine transporter (DAT) in the brain [22]. Thus, the more gradual rise of plasma MPH concentration with the OROS formulation of MPH could lead to a slower onset of blockade of the presynaptic DAT and therefore a lower risk for detection of euphoria [22]. Spencer et al [23] compared the relationship between peripheral and central pharmacokinetic properties of IR and OROS-MPH and their impact on abuse liability potential using C-11 altropane and positron emission tomography (PET) and found further support of this hypothesis.

The results of this study should be viewed in light of methodological limitations. Our criteria for ADHD were somewhat stricter than that DSM-IV. We required full childhood onset, whereas DSM-IV only requires some significant symptoms in childhood. The diagnosis of ADHD and assessment of ADHD symptoms also relied on self-report. Although the usefulness of self reports of ADHD symptoms are limited in pediatric samples, there is evidence that self reports of adults with ADHD correspond very well to corroborating histories provided by parents and spouses [24]. Also, assessment of adverse effects relied on spontaneous reports and such open-ended questioning of adverse events limits the precision of characterization that may be found in structured rating scales. While the current study is reassuring, strong inferences about tolerability and safety that require a much larger sample size to fully assess the occurrences of rare adverse events. Future work that can directly assess any improvements in treatment compliance associated with once-daily treatments for ADHD are also needed.

## Conclusion

Despite these limitations, comparison of data from two similarly designed, large, randomized, placebo-controlled trials showed that OROS-MPH has similar efficacy and tolerability as that of IR-MPH administered three times per day. These results indicate that MPH is highly effective for the treatment of adults with ADHD when delivered in appropriate doses and dosed across the day. More work is needed to evaluate whether these short-term benefits extend to the long-term.

## Competing interests

Dr. Joseph Biederman receives/d research support from, is/has been a speaker for, or is/has been on the advisory board for the following sources: Shire, Eli Lilly, Pfizer, McNeil, Abbott, Bristol-Myers-Squibb, New River Pharmaceuticals, Cephalon, Janssen, Novartis, UCB Pharma, Astra-Zeneca, Forest Laboratories, Glaxo-SmithKline, Neurosearch, Stanley Medical Institute, Inc, Lilly Foundation, Prechter Foundation, the National Institute of Health (NIH), National Institute of Mental Health (NIMH), National Institute of Child and Human Development (NICHD) and the National Institute on Drug Abuse (NIDA).

Dr. Eric Mick receives/d grant support is/has been a speaker for, or is/has been on the advisory board for the following sources: McNeil Pediatrics and Janssen Pharmaceuticals, Pfizer, Shire and the National Institute of Mental Health (NIMH).

Dr. Craig Surman has received research support from McNeil Pharmaceuticals, is on the speaker's bureau for Novartis Pharmaceuticals, and served in an advisory capacity to Shire Pharmaceuticals and Takeda Pharmaceuticals.

Dr. Thomas Spencer receives/d research support from, is/has been a speaker for, or is/has been on the advisory board for the following sources: Shire Laboratories, Inc, Eli Lilly & Company, Glaxo-Smith Kline, Pfizer Pharmaceutical, McNeil Pharmaceutical, Wyeth Ayerst, Pfizer Pharmaceutical, Novartis Pharmaceutical, and NIMH

Robert Doyle MD; Paul Hammerness MD; Evan Michel BA; Jessica Martin, MA declare they have no conflicts of interest.

## Authors' contributions

**JB: **Had full access to all the data in the study and take responsibility for the integrity of the data and the accuracy of the data analysis. I am also responsible for editing the final draft. I have read and approved the final manuscript. **EOM: **has made substantial contributions to conception and design, or acquisition of data, or analysis and interpretation of data, was involved in drafting the manuscript or revising it critically for important intellectual content, and I have read and approved the final manuscript. **CS: **contributed significantly to data acquisition. I have read and approved the final manuscript. **RD: **contributed significantly to data acquisition. I have read and approved the final manuscript. **PH: **contributed significantly to data acquisition. I have read and approved the final manuscript. **EM: **provided assistance in editing the manuscript for the intellectual content and accuracy of data described throughout the submission. I have read and approved the final manuscript. **JM: **contributed significantly to data acquisition. Provided my final approval of the version to be published. I have read and approved the final manuscript. **TS: **has made substantial contributions to conception and design, or acquisition of data, or analysis and interpretation of data. Have been involved in drafting the manuscript or revising it critically for important intellectual content. I have read and approved the final manuscript.

## Pre-publication history

The pre-publication history for this paper can be accessed here:


